# New Appliances and Things Medical

**Published:** 1897-07-10

**Authors:** 


### NEW APPLIANCES AND THINGS MEDICAL.

[We shall be glad to receive, at our Office, 28 & 29, Southampton Street, Strand, London, W.O., from the manufacturers, specimens of all new preparations and appliances which may be brought out from time to time.

### WHOLESOME SAUSAGES.

(Messrs. Radcliffe and Co., Heckmondwike.)

Cambridge sausages are of world-wide fame. But under the title of "Cambridge" many very inferior sausages are purchased to the discredit of the better sorts. It is therefore essential to find a reliable emporium. We can confidently recommend Messrs. Radcliffe's as such. Their sausages are most carefully made, the ingredients of the best quality, and the flavour excellent. Really well-made sausages such as sold by Messrs. Radcliffe are most useful adjuncts to the larder, owing to their excellent keeping qualities. This we have tested with the best results. The sausage when cold has hardly yet been appreciated as it should, seeing that it forms one of the most nourishing and convenient forms of portable food for the bicyclist or pedestrian on tour. Cambridge sausages when cooked will remain perfectly good for many days in the hottest weather. They are an excellent "reserve" carried in a small box or tin. The cost is so small that if not required the waste is not worth considering in comparison with the condition of independence of wayside catering which it secures.

